# Development of *Dirofilaria immitis* and *Dirofilaria repens* in *Aedes japonicus* and *Aedes geniculatus*

**DOI:** 10.1186/s13071-017-2015-x

**Published:** 2017-02-20

**Authors:** Cornelia Silaghi, Relja Beck, Gioia Capelli, Fabrizio Montarsi, Alexander Mathis

**Affiliations:** 1 National Centre for Vector Entomology, Institute of Parasitology, Vetsuisse Faculty, University of Zurich, Zurich, Switzerland; 2 Department for Bacteriology and Parasitology, Croatian Veterinary Institute, Zagreb, Croatia; 3 Laboratory of Parasitology, Istituto Zooprofilattico Sperimentale delle Venezie, Legnaro, Italy

**Keywords:** *Dirofilaria immitis*, *Dirofilaria repens*, *Aedes japonicus*, *Aedes geniculatus*, *Aedes aegypti*, Microfilariae, Dog, Vector, Intermediate host

## Abstract

**Background:**

The mosquito-borne filarial nematodes *Dirofilaria immitis* and *Dirofilaria repens* primarily affect dogs but also cats, causing heartworm disease or subcutaneous dirofilariosis, respectively, and both may also cause zoonotic diseases in humans. Several mosquito species have been reported as competent vectors for these nematodes, but no data are available for the invasive mosquito species *Aedes japonicus* (Theobald, 1901). The objective of this study was to describe the development of both *D. immitis* and *D. repens* under standardised experimental laboratory conditions in mosquitoes.

**Methods:**

For this purpose, both a laboratory strain and field-collected individuals of the invasive mosquito species *Ae. japonicus* and, for comparative purposes, a laboratory strain of *Aedes geniculatus*, a rare indigenous species sharing habitats with *Ae. japonicus*, and of the tropical species *Aedes aegypti* were used. Anticoagulated microfilariaemic blood was fed at a density of 3000 mf/ml to mosquitoes with a hemotek system. Blood-fed mosquitoes were incubated at 27 °C and 85% relative humidity, and specimens were dissected under the microscope at pre-set time points to observe developmental stages of both *Dirofilaria* species. Additionally, real-time PCRs were carried out in some microscopically negative samples to determine the infection rates.

**Results:**

In field-collected *Ae. japonicus* infectious L3 larvae of both *D. immitis* and *D. repens* developed, rendering this mosquito species an efficient vector for both filarial species. Additionally, *Ae. geniculatus* was shown to be an equally efficient vector for both filarial species. *Aedes japonicus* mosquitoes from a laboratory colony were refractory to *D. immitis* but susceptible to *D. repens*, whereas *Ae. aegypti* was refractory to both filarial species.

**Conclusions:**

To our knowledge, *Aedes japonicus* was for the first time shown to be an efficient vector for both *D. immitis* and *D. repens*, indicating that this invasive and locally highly abundant species may contribute to a transmission of filarial worms. The data emphasize the necessity to perform vector competence studies with local mosquito populations as basis for risk assessments. We further demonstrated that detection of filarial DNA in a mosquito species alone does not allow to draw reliable conclusions with regard to its vector competence.

## Background

The mosquito-borne filarial nematodes *Dirofilaria immitis* (Leidy, 1856) and *Dirofilaria repens* (Railliet & Henry, 1911) primarily affect dogs but also cats, causing cardiopulmonary (heartworm disease) or subcutaneous dirofilariosis, respectively. Both filarial worms may also cause zoonotic diseases in humans, in the form of pulmonary (*D. immitis*) or subcutaneous/ocular (*D. repens*) dirofilariosis [1, 2]. Both species have expanded their distribution range in the recent past [1, 3]. *Dirofilaria immitis* is endemic globally in regions with tropical or subtropical climates, whereas *D. repens* is restricted to the Old World. Recent detections of DNA of the canine heartworm in mosquitoes [4–6] in temperate climate areas in central Europe as well as climate analyses [4, 7–9] suggest a northward spread. *Dirofilaria repens* seems to be already endemic in central Europe, based on several DNA detections in mosquito populations [5, 10–12] and a growing number of autochthonous cases in both dogs and humans [13–20].

The transmission of the filarial worms is dependent on the availability of microfilariaemic hosts, competent mosquito vectors and suitable temperatures for the development of the infectious stages in the mosquito [8]. A major driver for the range expansion of canine dirofilarioses is the transport of infected animals from endemic to new areas, e.g. *via* the import of dogs from Mediterranean countries to central Europe where several mosquito species occur which have a demonstrated vector competence for *Dirofilaria* spp. [21]. Transmission is feasible in regions where suitable temperatures allow the development of the microfilariae (mf) to the infectious third larval stage (L3) which migrate to the proboscis of the mosquito. This extrinsic development is possible above 14 °C and is completed when the sum of the daily average degrees above this threshold value has reached at least 130. This value was initially termed ‘heartworm development unit’ (HDU) but later adapted to ‘*Dirofilaria* development unit’ (DDU) due to similar temperature requirements for both *Dirofilaria* species [22, 23]. Thus, the extrinsic development takes e.g. 10–12 days at 24–26 °C but as long as 29 days at 18 °C. Successful transmission of filariae to a host requires an infected mosquito to survive longer than the duration of the extrinsic incubation period. Assuming a maximal life span of 30 days for a mosquito, models revealed that summer temperatures allow the development of L3 at latitudes of 50° N in Europe [23]. Indeed, a canine autochthonous case of *D. immitis* dirofilariosis was observed at 54° N [24].

In addition, during recent decades, invasive container-breeding aedine mosquito species have been recorded in areas of Europe [25], and they might contribute to an increased transmission risk of *Dirofilaria* spp. as evidenced for the Asian tiger mosquito *Aedes albopictus* (Skuse, 1894) (= *Stegomyia albopicta*) in Italy [26, 27]. Local populations of another invasive species, *Aedes koreicus* (Edwards, 1917) (= *Hulecoeteomyia koreica*), have been recorded in few instances [28–30], and this species was shown in an experimental study to allow the development of the infectious L3 larvae of *D. immitis* [31].

No data with regard to vector competence for *Dirofilaria* spp. are available for a third invasive mosquito species, *Aedes japonicus* (Theobald, 1901) (= *Hulecoeteomyia japonica*). This East Asian native mosquito has in recent years invaded large parts of North America and many countries in Europe [32, 33], and it is further expanding [34, 35] (for updated European maps see www.ecdc.europa.eu).

The objectives of this study were to describe the development of both *D. immitis* and *D. repens* under standardised experimental laboratory conditions in both a laboratory strain and in field-collected individuals of the invasive mosquito species *Ae. japonicus*. For comparative purposes, these experiments were also done with each a laboratory strain of *Aedes geniculatus* (Olivier, 1791) (= *Dahliana geniculata*), a rare species in the Palaearctic Region sharing habitats with *Ae. japonicus*, and of the tropical species *Aedes aegypti* (L.) (= *Stegomyia aegypti*).

## Methods

### Microfilariae inoculum

Presence, vitality and number of microfilariae in all samples obtained from dogs (see below) were confirmed by microscopy. Briefly, 20 μl of blood were mixed with 40 μl of distilled water, covered with a cover slide, and microfilariae were counted by examination with a microscope under 100× magnification. Microfilaraemiae of the dogs were determined as average from three counts. Blood was anticoagulated with EDTA or heparin.

#### *Dirofilaria immitis*

Microfilariaemic blood samples from experimentally infected dogs (field isolate from northern Italy) as well as blood from uninfected dogs were kindly provided by Christian Epe (Elanco, St. Aubin, Switzerland).

#### Dirofilaria repens

Blood samples were from a dog naturally infected with *D. repens* (selected by one of the authors, RB). The infected dog was a mixed breed, 4.5 year-old and had never left the Daruvar region in north-eastern Croatia. The dog had first been diagnosed positive for microfilariae 2 years prior to the experiments. He was regularly checked by a local veterinarian and was always clinically unremarkable (nice fur, healthy skin, no nodules observed). The infection with *D. repens* was confirmed on DNA from the blood samples by a conventional PCR [36]. No co-infections with other filariae that are covered with this diagnostic approach (*Acanthocheilonema reconditum*, *Acanthocheilonema, Dracunculoides* spp., *Brugia pahangi*, *Brugia malayi*, *D. immitis*) were detected.

### Mosquitoes and inoculation

#### Field-collected *Aedes japonicus*

Host-seeking female *Ae. japonicus* were collected in a forest area within the urban borders of the city of Zurich, using mouth aspirators with four persons (“human baits”). Dry ice was additionally used as attractant. Mosquitoes were transferred into a cylindrical 500 ml plastic cage with moist cotton wool and were taken to the laboratory within 1 h of collection in the field for oral inoculation.

#### Laboratory strains

Laboratory colonies of *Ae. japonicus* (Pennsylvania strain, PEN) and *Ae. aegypti* (IPNC) were reared and maintained in a climate chamber in an insectarium under standard laboratory conditions at a temperature of 24 °C (*Ae. japonicus*) or 27 °C (*Ae. aegypti*), a relative humidity (rh) of 85% and a light-dark cycle of 16:8 h including dusk/dawn phases of 1 h. A recently established colony of *Ae. geniculatus* (IPZ) [37] was maintained at room temperature as described. Mosquitoes were provided with 5% glucose solution and water *ad libitum*. For reproduction they were either provided a mouse as blood source once a week (*Ae. japonicus*, *Ae. aegypti*; approved by the Cantonal Veterinary Office of Zurich, permission number ZH064/15) or sheep blood (approved by the Cantonal Veterinary Office of Zurich, permission number ZH008/15) using a standard artificial feeding system (Hemotek^TM^, Hemotek Ltd, Lancashire, UK). For the inoculation experiments, females at an age of 5–7 days were chosen.

#### Oral inoculation of mosquitoes

Microfilariaemic counts were adjusted to 3000 mf/ml with blood from uninfected dogs. Sugar was removed and the mosquitoes were allowed to feed through Parafilm® membranes for at least 2 h on 2 ml blood at 37 °C in a Hemotek^TM^ system. In order to boost the blood feeding rates of the field-collected mosquitoes, adenosine triphosphate (ATP, final concentration 5 mM) was added to the blood [38], and iGu® lure disks (Combi FRC 3003, Silva GmbH & Co. KG, Lübeck, Germany) were displayed. Mosquitoes that did not take a full blood meal were discarded.

#### Maintenance of the mosquitoes after inoculation

Mosquitoes were kept for up to 21 days in a double containment (inner cage 17 × 17 cm, outer cage 32.5 × 32.5 cm, both Bugdorm, MegaView Science, Taichung, Taiwan) in an incubator at 27 °C/85% rh with access to 5% glucose and water *ad libitum*.

### *Dirofilaria* detection in mosquitoes

#### Microscopic investigation

Mosquitoes were anaesthetised by brief exposure to CO_2_ supplied by dry ice and immobilised by removing their wings and legs on a chill table. Afterwards, they were kept at 4 °C and dissected individually and immediately before microscopic examination. Malpighian tubules and midgut were separated from the abdomen, and afterwards abdomen, head and thorax were dissected on separate microscopic slides. Clean entomological forceps and sterile needles were used for each dissection. A drop of phosphate buffered saline (PBS) was added onto each slide and the sample carefully covered with a coverslide. Light pressure was applied to facilitate the detection of worms, especially of the early stages of development [39]. All slides were investigated with differential interference contrast microscopy (DIC) with a Leica DM 6000 B microscope at 100–400× magnification. Photographs of larval stages were taken and their length and width calculated. The Leica software LAS was used for all the analyses.

Within 24 h of infection, up to three mosquitoes of each group were dissected to assess the number of microfilariae ingested per blood meal. Afterwards, up to three mosquitoes were dissected at fixed days (see below) to investigate the developmental stages of the larvae according to size and morphology [39, 40]. Freshly dead mosquitoes were also dissected; mosquitoes already dead for a while and desiccated were subjected to PCR analysis only (see below). After microscopic investigation, the slides were rinsed with a few drops of PBS for subsequent PCR analysis.

#### Molecular analysis

Mosquito samples with 180 μl TE buffer were disrupted in a TissueLyser II (Qiagen, Hilden, Germany) in a 2 ml Eppendorf tube with one 5 mm stainless steel bead added at 30 beats per second for 1 min with a centrifugation step after 30 s. The samples were incubated with lysis buffer and proteinase K for at least 4 h or overnight. Afterwards, DNA was isolated using the Qiamp DNA mini kit (Qiagen) according to the manufacturer’s instruction. DNA was eluted in 55 μl AE buffer and stored at -20 °C until further use.

A real-time PCR targeting the mitochondrial COX 1 gene [41] of *D. immitis* and *D. repens* was designed using GenScript (www.genscript.com/ssl-bin/app/primer). Forward and reverse primers and the Taqman® probe were as follows: Diro-f: 5′-GGT GTT TGG GAT TGT TAG TGA A-3′; Diro-r: 5′-CAG CAA TCC AAA TAG AAG CAA-3′; Diro-p: 5′-FAM-TCT GGC CAA ACA AAC GAT CCT TAT CA-TAMRA-3′. The target size is 98 bp. The PCR assay was not evaluated for its diagnostic value.

Real-time PCR was performed in addition to microscopical investigations with microscopically negative samples and with dead mosquitoes as follows: up to day 5 post-infection (dpi) with abdomens only, after 7 dpi on all negative abdomens, thoraces and heads. Pools were used if there were more than three samples of the same kind on the same day of infection.

#### Data analysis

For each trial, feeding rates as well as mortality rates at 1 and 5 dpi and at 14 dpi were calculated. Differences in mortality rates within and between species were calculated in a 2 × 2 contingency table using Fisher’s exact test with two-tailed *P*-values on GraphPad Software (www.graphpad.com); *P*-values below 0.05 were considered as statistically significant.

The positivity rate of the mosquitoes was calculated as the percentage of blood-fed mosquitoes that had any developmental stage and/or positive PCR result out of all blood-fed mosquitoes.

The following indices of experimental filarial infections were calculated based on previous publications [31, 42]

$$ \mathrm{Infection}\ \mathrm{rate}\ \mathrm{I}\mathrm{R}=\frac{number\  of\  blood- fed\  mosquitoes\  with\  L3\  in\  body}{surviving\  mosquitos\  at\  end\  of\  in cubation\  period}\times 100 $$

$$ \mathrm{Vector}\ \mathrm{efficiency}\ \mathrm{index}\ \mathrm{V}\mathrm{E}\mathrm{I} = \frac{average\  number\  of\  L3\  in\  mosquitos\  from\  end\  of\  in cubation\  period\  to\  end\  of\  study}{average\  number\  of\  in gested\  microfilariae}\times 100 $$

The end of the incubation period was set at 10 dpi, as this equals the theoretical extrinsic incubation time at 27 °C according to DDUs [22]. If L3 larvae appeared before 10 dpi, calculations were based on the first appearance of L3.

Additionally to the above mentioned calculations, the first appearance of motile L3 in the proboscis was taken into account to assess the vector competence.

## Results

### Infection trial

Mosquitoes of laboratory strains of *Ae. japonicus* (*n* = 65), *Ae. geniculatus* (*n* = 35) and *Ae. aegypti* (*n* = 105) as well as field-collected *Ae. japonicus* (*n* = 151) were allowed to feed through Parafilm® membranes on blood containing microfilariae of *D. immitis* or *D. repens*, or on negative control blood (*Ae. japonicus* groups only) (Table 1). The feeding rates were around 50% for all *Ae. aegypti* and *Ae. japonicus* except in one treatment (24%, field-collected *Ae. japonicus* feeding on *D. repens* blood, Table 1), and around 70% for the *Ae. geniculatus* groups.

**Table 1:** Feeding and mortality rates of *Aedes japonicus* (laboratory strain PEN; field-collected specimens from Switzerland (CH), *Ae. geniculatus* (laboratory strain IPZ) and *Ae. aegypti* (laboratory strain IPNC) during infection trials with *Dirofilaria immitis* and *D. repens*

Mosquito species	Inoculation	No. total	No. feeding (%)	Mortality^a^ at 1 dpi No. (%)	Mortality^a^ 5 dpi No. (%)	Mortality^a^ at 14 dpi No. (%)
*Ae. japonicus* (PEN)	*Dirofilaria repens*	19	8 (42.1)	0	0	2 (28.6)
*Ae. japonicus* (PEN)	*D. immitis*	28	12 (42.3)	0	5 (50.0)	8 (80.0)
*Ae. japonicus* (PEN)	Negative control	18	9 (50.0)	0	3 (33.3)	4 (44.4)
*Ae. japonicus* (CH)	*D. repens*	72	17 (23.6)	0	0	0
*Ae. japonicus* (CH)	*D. immitis*	60	31 (51.6)	0	10 (35.7)	13 (46.4)
*Ae. japonicus* (CH)	Negative control	19	8 (42.1)	0	1 (12.5)	5 (62.5)
*Ae. geniculatus* IPZ	*D. repens*	17	12 (70.1)	0	0	4 (44.4)
*Ae. geniculatus* IPZ	*D. immitis*	18	13 (72.2)	0	2 (18.2)	4 (36.4)
*Ae. aegypti* IPNC	*D. repens*	46	21(45.7)	4 (22.2)	8 (44.4)	8 (44.4)
*Ae. aegypti* IPNC	*D. immitis*	41	24 (58.5)	6 (28.6)	11 (52.4)	11 (52.4)

^a^Only mosquitoes that died naturally; calculated without day 1 samples; numbers are cumulative from day 1

Cumulative mortality rates of naturally dead mosquitoes were calculated from the total number of blood-fed mosquitoes in comparison to those that died until 1 and 5 dpi and until 14 dpi. Mosquitoes taken alive for dissection were not considered as dead mosquitoes but were assumed to have lived until 14 dpi (Table 1). At 1 dpi, mortality only occurred in the *Ae. aegypti* IPNC group; this was statistically significantly different to the mortality in field-collected *Ae. japonicus* infected with *D. immitis* (*P* = 0.046). No other statistically significant differences were observed within the *D. immitis* experiments between the different species at 5 and at 14 dpi.

Until 5 dpi, there was no mortality in the *D. repens* inoculated groups, except in *Ae. aegypti* IPNC. The mortality on 5 dpi in the *D. repens* inoculated groups was significantly different between *Ae. aegypti* IPNC and *Ae. geniculatus* (*P* = 0.0299) and field collected *Ae. japonicus* (*P* = 0.0047). Mortality rates varied between 0 and 80%, and were usually around 40% or higher at 14 dpi, including the control mosquitoes. The exception was the field-collected *Ae. japonicus* population inoculated with *D. repens* with an overall mortality rate of 0%. This was significantly different to the mortality in other *D. repens* infected groups (*P* = 0.0361 for *Ae. japonicus* PEN, *P* = 0.0211 for *Ae. geniculatus* and *P* = 0.01 for *Ae. aegypti*) and significantly different in comparison to this field population infected with *D. immitis* at 5 dpi and at 14 dpi (*P* = 0.0086 and 0.0035, respectively). Only in *Ae. aegypti* IPNC was the overall mortality rate observed already at 5 dpi, whereas in all other mosquito species mortality increased until at 14 dpi (Table 1).

The calculated infectious dose per mosquito in the trials was 12 mf, considering the microfilarial density of 3000/ml blood and an average blood meal volume of 4 μl. The observed infectious doses as determined by microscopy at 1 dpi differed considerably (Tables 2, 3, 4, 5, 6, 7, 8 and 9). For *D. repens* they varied from 0 to 22 and for *D. immitis* from 0 to 7.

**Table 2:** Specimens of laboratory colony *Aedes japonicus* PEN (*n* = 10) examined microscopically for larval stages of *Dirofilaria immitis*

	Mosquitoes		Number of larval stages per individual positive mosquito				
Dpi	Total no.	No. positive	mf	L1	L2	L3	Location (total no. in all dissected mosquitoes)^b^
1	2	1^a^	2	–	–	–	Midgut (2)
3	3	2^a^	–	1, 3	–	–	Malpighian tubules (4)
4	1	0^a^	–	–	–	–	
5	2	1^a^	1	11	–	–	Malpighian tubules (11, of which 3 melanized)
6	1	0^a^	–	–	–	–	
14	1	0	–	–	–	–	

*Abbreviations*: *dpi* day post-inoculation, *mf* microfilariae, *L1* first-stage larva, *L2* second-stage larva, *L3* third-stage larva (infectious stage)

^a^One additional mosquito positive in PCR only

^b^Data given only when localisation was clearly assignable after dissection

**Table 3:** Specimens of laboratory colony *Aedes japonicus* PEN (*n* = 8) examined for larval stages of *Dirofilaria repens*

	Mosquitoes		Number of larval stages per individual positive mosquito				
Dpi	Total no.	No. positive	mf	L1	L2	L3	Location (total no. in all dissected mosquitoes)^a^
1	1	1	22	–	–	–	
3	1	1	1	–	–	–	
5	1	1	–	1	–	–	
6	2	1	–	1	–	–	
12	1	0	–	–	–	–	
14	1	1	–	–	–	8	Proboscis (6), head (1), Malpighian tubules (1)
16	1	1	–	–	–	4	Head (1), abdomen (3)

*Abbreviations*: *dpi* day post-inoculation, *mf* microfilariae, *L1* first-stage larva, *L2* second-stage larva, *L3* third-stage larva (infectious stage)

^a^Data given only when localisation was clearly assignable after dissection

**Table 4:** Specimens of *Aedes japonicus* (CH) (*n* = 24) examined microscopically for larval stages of *Dirofilaria immitis*

	Mosquitoes		Number of larval stages per individual positive mosquito				
Dpi	Total no.	No. positive	mf	L1	L2	L3	Location (total no. in all dissected mosquitoes)^c^
1	3	2	5, 4	–	–	–	
3	6	6	1, 1, 1, 1	5, 4, 2, 1	–	–	
4	3	1^b^	–	6	–	–	
7	3	2	–	7	5, 3	1	In Malpighian tubules, L1 melanized
10	3	2	–	–	–	1, 6	In Malpighian tubules (7)
14	3^a^	3	–	–	1	1, 3	Head (1), Malpighian tubules (2)
21	3	3	–	–	1	1, 4, 9	Proboscis (7), head (1), thorax (5)

*Abbreviations*: *dpi* day post-inoculation, *mf* microfilariae, *L1* first-stage larva, *L2* second-stage larva, *L3* third-stage larva (infectious stage)

^a^Proboscis of one mosquito was lost during the dissection process

^b^Additional two positive in PCR only

^c^Data given only when localisation was clearly assignable after dissection

**Table 5:** Specimens of *Aedes japonicus* (CH) (*n* = 17) examined for larval stages of *Dirofilaria repens*

	Mosquitoes		Number of larval stages per individual positive mosquito				
Dpi	Total no.	No. positive	Mf	L1	L2	L3	Location (total no. in all dissected mosquitoes)^a^
1	5	4	19, 20, 8, 1	–	–	–	
4	2	2	–	11, 18	–	–	
7	2	2	–		23, 9	–	L2 in Malpighian tubules (32)
10	3	3	–	–	4, 1	6, 3, 12	Proboscis (9), thorax (9), abdomen (4), Malpighian tubules (4)
14	3	3	–	1, 5	7	8, 11, 11	proboscis (26), head (1), abdomen (3)
21	2	2	–	–	–	12, 3	Proboscis (13), abdomen (2)

*Abbreviations*: *dpi* day post-inoculation, *mf* microfilariae, *L1* first-stage larva, *L2* second-stage larva, *L3* third-stage larva (infectious stage)

^a^Data given only when localisation was clearly assignable after dissection

**Table 6:** Specimens of laboratory colony *Aedes geniculatus* IPZ (*n* = 9) examined microscopically for larval stages of *Dirofilaria immitis*

	Mosquitoes		Number of larval stages per individual positive mosquito				
Dpi	Total no.	No. positive	Mf	L1	L2	L3	Location (total no. in all dissected mosquitoes)^c^
1	2	2	5, 6	–	–	–	
4	2	0^a^	–	–	–	–	
7	2	2^b^	–	25	–	–	Malphighian tubules (24), melanized in Malpighian tubules (1)
9	0	0^b^	–	–	–	–	
10	2	1^b^	–	–	–	11	Malpighian tubules (11)
13	1	1	–	–	–	6	Abdomen (5), Thorax (1)

*Abbreviations*: *dpi* day post-inoculation, *mf* microfilariae, *L1* first-stage larva, *L2* second-stage larva, *L3* third-stage larva (infectious stage)

^a^Two additional positive in PCR only

^b^One additional positive in PCR only

^c^Data given only when localisation was clearly assignable after dissection

**Table 7:** Specimens of laboratory colony *Aedes geniculatus* IPZ (*n* = 8) examined microscopically for larval stages of *Dirofilaria repens*

	Mosquitoes		Number of larval stages per individual positive mosquito				
Dpi	Total no.	No. positive	Mf	L1	L2	L3	Location (total no. in all dissected mosquitoes)^a^
1	3	3	6, 8, 20	–	–	–	
5	2	2	–	3, 7	–	–	Malpighian tubules (3)
9	1	1	–	3	19	14	Malphighian tubules (36)
14	2	2	–	1	–	1, 25	L3: Proboscis (20), thorax (2), Malpighian tubules (4)

*Abbreviations*: *dpi* day post-inoculation, *mf* microfilariae, *L1* first-stage larva, *L2* second-stage larva, *L3* third-stage larva (infectious stage)

^a^Data given only when localisation was clearly assignable after dissection

**Table 8:** Specimens of laboratory colony *Aedes aegypti* IPNC (*n* = 22) examined microscopically for larval stages of *Dirofilaria immitis*

			Number of larval stages per individual positive mosquito^c^			
Dpi	Total no. of individuals	No. of positive individuals	mf	L1	L2	L3
1	9	5^a^	1, 1, 5, 7, 10	–	–	–
2	1	1	–	1	–	–
3	4	1^b^	1	–	–	–
5	2	1	–	3	–	–
7	2	0^a^	–	–	–	–
9	1	0^b^	–	–	–	–
12	1	0^b^	–	–	–	–
14	1	0^b^	–	–	–	–
16	1	0^b^	–	–	–	–

*Abbreviations*: *dpi* day post-inoculation, *mf* microfilariae, *L1* first-stage larva, *L2* second-stage larva, *L3* third-stage larva (infectious stage)

^a^Two additional positive in PCR

^b^One additional positive in PCR only

^c^Localisation of developmental stages was not clearly assignable after dissection

**Table 9:** Specimens of laboratory colony *Aedes aegypti* IPNC (*n* = 18) examined microscopically for larval stages of *Dirofilaria repens*

			Number of larval stages per individual positive mosquito^c^			
Dpi	Total no. of individuals	No. of positive individuals	Mf	L1	L2	L3
1	6	3^a^	1, 3, 6	–	–	–
3	4	2	16, 2	–	–	–
5	2	1	–	1	–	–
7	2	0^a^	–	–	–	–
9^b^	1	0^a^	–	–	–	–
12	1	0	–	–	–	–
14	1	0	–	–	–	–
16	1	0	–	–	–	–

*Abbreviations*: *dpi* day post-inoculation, *mf* microfilariae, *L1* first-stage larva, *L2* second-stage larva, *L3* third-stage larva (infectious stage)

^a^One additional positive in PCR only

^b^Head was lost during the dissection process

^c^Localisation of developmental stages was not clearly assignable after dissection

### *Aedes japonicus* (PEN) inoculated with *D. immitis* or *D. repens*

**Fig. 1:**
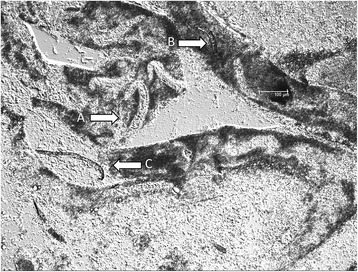
Alive (**a**) and melanised (**b**) first-stage larvae (L1) and melanised microfilaria (**c**) of *Dirofilaria immitis* in Malpighian tubules of *Aedes japonicus* PEN at 5 dpi

Altogether 12 blood-fed mosquitoes were in the trial with *Ae. japonicus* PEN inoculated with microfilariae of *D. immitis*. Out of these, alive mosquitoes (*n* = 4) or freshly dead mosquitoes (*n* = 6) were dissected (Table 2), and a further 2 dead mosquitoes (desiccated) were analysed by PCR only. Four of the 10 dissected mosquitoes were positive for *D. immitis* in microscopy, and another 5 were positive by PCR only. The 2 dead and desiccated mosquitoes were PCR positive [at 2 dpi (abdomen) and 12 dpi (abdomen and head)]. Thus, 11 out of the total 12 mosquitoes (91.7%) were positive for *D. immitis*. Mosquitoes were positive in microscopy until at 5 dpi, but by PCR the last positive was found at 12 dpi. Figure 1 shows L1 larvae (alive and melanized) in the Malpighian tubules and a melanized microfilaria at 5 dpi. As no L3 of *D. immitis* were observed, both IR and VEI for *D. immitis* in *Ae. japonicus* PEN were 0%. However, the head of a dead mosquito was PCR-positive at 12 dpi. Nonetheless, *Ae. japonicus* PEN may be refractory to infection with *D. immitis*

Eight blood-fed mosquitoes were in the trial with *Ae. japonicus* PEN inoculated with microfilariae of *D. repens* (Table 3), and all mosquitoes were dissected (6 alive and 2 freshly dead). Six of the 8 mosquitoes were positive for *D. repens* in microscopy (75%), and no additional one was identified positive by PCR. First L3 larvae were observed at 14 dpi in abdomen and proboscis (Table 3). The IR for *D. repens* was calculated to be 33.3% and the VEI 18.2%. Thus, *Ae. japonicus* PEN is susceptible to infection with *D. repens*.

### *Aedes japonicus* CH inoculated with *D. immitis* or *D. repens*

**Fig. 2:**
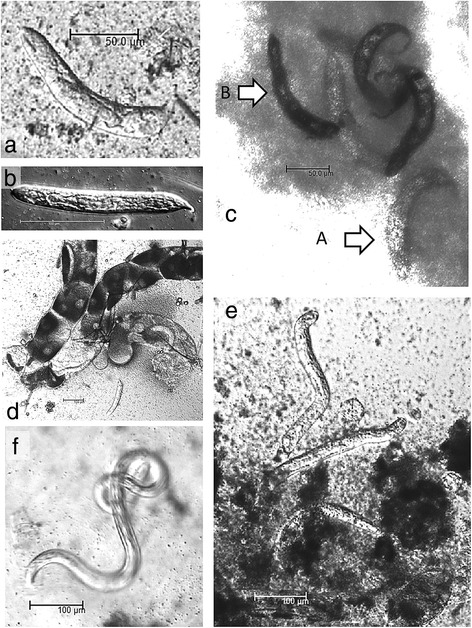
Developmental stages of *Dirofilaria immitis* in *Aedes japonicus* collected from the field in Switzerland: **a**, **b** L1 larvae at 4 dpi. **c** L1 alive (**A**) and melanised (**B**) larvae at 7 dpi. **d** L1 larvae in Malpighian tubules at 4 dpi. **e** L2 larvae at 7 dpi. **f** L3 larvae at 14 dpi

Thirty-one blood-fed mosquitoes were in the trial with *Ae. japonicus* CH inoculated with microfilariae of *D. immitis*, and 24 mosquitoes were dissected (18 alive and 6 freshly dead; see Table 4). Nineteen were positive for *D. immitis*, and an additional 2 were identified positive by PCR. Five of 7 further dead and desiccated mosquitoes were positive for *D. immitis* DNA in their abdomens at 2, 7, 8 and 10 dpi. Thus, altogether 26 out of total 31 (83.9%) mosquitoes were positive for *D. immitis*. Various developmental stages are shown in Fig. 2.

**Fig. 3:**
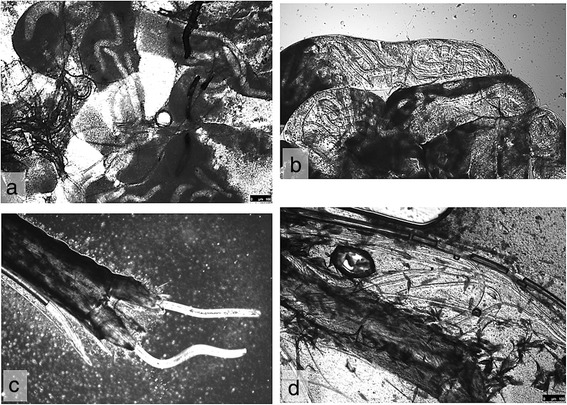
Developmental stages of *Dirofilaria repens* in *Aedes japonicus* collected from the field in Switzerland: **a** Different developmental stages, alive and melanized, in Malpighian tubules at 14 dpi. **b** L2, and possibly L3 larvae, in Malpighian tubules at 7 dpi. **c**, **d** L3 larvae at 14 and 21 dpi, respectively

Seventeen mosquitoes took a blood meal containing microfilariae of *D. repens* (Table 5). There were no mortalities, and all were dissected. A total of 16 (94.1%) were positive for *D. repens*. The only microscopically negative mosquito was also negative by PCR. Different developmental stages are shown in Fig. 3.

First L3 larvae of *D. immitis* were observed at 14 dpi in the proboscis and of *D. repens* at 10 dpi. L3 of both *Dirofilaria* species were found until the end of the experiments at 21 dpi.

The IR and VEI were 27.8 and 66.7% for *D. immitis*, and 47.1 and 85.9% for *D. repens*, respectively, rendering *Ae. japonicus* CH a susceptible vector for both filarial species.

### *Aedes geniculatus* IPZ inoculated with *D. repens* or *D. immitis*

Thirteen blood-fed mosquitoes were in the trial with *Ae. geniculatus* IPZ inoculated with microfilariae of *D. immitis*, and 9 alive mosquitoes were dissected (Table 6). Six of them were positive in microscopy for *D. immitis*, and an additional 3 were identified positive by PCR. Furthermore 3 of 4 dead and desiccated mosquitoes was positive for *D. immitis* DNA in their abdomens at 4, 7 and 9 dpi. Thus, altogether 12/13 (92.3%) mosquitoes were positive for *D. immitis*.

**Fig. 4:**
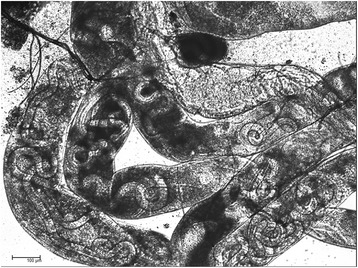
L2 and L3 larvae of *Dirofilaria repens* in Malpighian tubules of *Aedes geniculatus* (laboratory strain IPZ) at 9 dpi

Twelve mosquitoes took a blood meal containing microfilariae of *D. repens*. Eight were dissected and were positive in microscopy (Table 7). One out of 4 dead and desiccated mosquitoes was also PCR positive. Thus, 9 out of 12 (75%) mosquitoes were positive for *D. repens*. Figure 4 shows a massive infection of Malpighian tubules with L2 and L3 larvae at 9 dpi.

First L3 larvae of *D. immitis* were observed at 10 dpi in the abdomen and at 13 dpi also in the thorax. *Dirofilaria repens* L3 first occurred at 9 dpi in Malpighian tubules and at 14 dpi in the proboscis.

The IR and VEI were 22.2 and 100% for *D. immitis*, and 37.5 and 70.8% for *D. repens*, respectively. Thus, *Ae. geniculatus* IPZ is susceptible to both filarial species.

### *Aedes aegypti* IPNC inoculated with *D. repens* or *D. immitis*

Altogether 24 blood-fed mosquitoes were in the trial with *Ae. aegypti* IPCN inoculated with microfilariae of *D. immitis* (Table 8), and 22 (14 alive and 8 freshly dead) were dissected. Eight were positive for *D. immitis* in microscopy and an additional 9 were positive by PCR on DNA from the abdomens (up to 16 dpi). Two further dead and desiccated specimens were negative by PCR. Therefore, a total of 17/24 (70.8%) mosquitoes were positive.

Nineteen blood-fed mosquitoes were in the trial with *Ae. aegypti* IPCN inoculated with microfilariae of *D. repens* (Table 9) and 18 (12 alive and 6 freshly dead) were dissected. Six were positive for *D. repens* in microscopy and an additional 3 by PCR (up to day 9). A total of 9 out of 19 (47.4%) mosquitoes were positive.

Microfilariae and L1 larvae could only be observed in microscopy until at 5 dpi, and no developmental stages were observed after day 5 for both filarial species. As no L3 developed, infection rate and vector efficiency index were 0% for both *D. immitis* and *D. repens*. Therefore, according to our study, *Ae. aegypti* IPNC is refractory to infection with both filarial species.

### Development of larval stages

Infected Malpighian tubules had sac-like appearance and developmental stages were generally found in the distal part of the tubules, as has been described previously [22]. In none of the mosquitoes were all tubules infected. The length and width of the observed larval stages in the mosquitoes are shown in Tables 10 and 11. Generally, fairly large variations in the sizes of the developmental stages were observed. Additionally, melanized larval stages were observed: for *D. immitis* in *Ae. japonicus* PEN at 5 dpi (microfilaria: 305 × 8 μm; L1: 164 × 22 μm) and in *Ae. japonicus* CH at 7 dpi (L1: 157 × 24 μm; 173 × 24 μm; 187 × 25 μm), and for *D. repens* in *Ae. japonicus* CH at 14 dpi (L1: 177 × 26 μm).

**Table 10:** Sizes of developmental stages of *Dirofilaria repens* reared under laboratory conditions at 27 °C

Days post-infection	Measurement (μm)	*Ae. japonicus* PEN	*Ae. japonicus* CH	*Ae. geniculatus* IPZ	*Ae. aegypti* IPNC
1–2	Mean length (range)	331 (309–350)	–	237 (221–254)	–
	Mean width (range)	8 (5–10)	–	6 (5–6)	–
3–6	Mean length (range)	208(144–272)	–	–	345 (313–361)
	Mean width (range)	25 (21–30)	–	–	7 (6–9)
7–9	Mean length (range)	263 (na)	602 (535–678)	732 (518–848)	–
	Mean width (range)	283 (na)	30 (23–34)	35 (10–48)	–
10–14	Mean length (range)	954 (822–1030)	822 (685–954)	911 (657–1120)	–
	Mean width (range)	26 (23–28)	24 (22–27)	29 (26–36)	–
More than 14	Mean length (range)	976 (na)	–	–	–
	Mean width (range)	27 (na)	–	–	–

*Abbreviations*: *na* not applicable (only single specimens available); –, no specimens available or no photographs taken during dissection process

**Table 11:** Sizes of developmental stages of *Dirofilaria immitis* reared under laboratory conditions at 27 °C

Day post-infection	Measurement (μm)	*Ae. japonicus* PEN	*Ae. japonicus* CH	*Ae. geniculatus* IPZ	*Ae. aegypti* IPNC
1–2	Mean length (range)	–	–	–	283 (256–290)
	Mean width (range)	–	–	–	8 (6–10)
4–6	Mean length (range)	166 (122–215)	218 (171–250)	–	260 (244–270)
	Mean width (range)	22 (14-35)	25 (23–28)	–	5.9 (5.5–6.2)
7–9	Mean length (range)	–	284 (170–353)	359 (283–418)	–
	Mean width (range)	–	28 (25–33)	32 (28–35)	–
10–14	Mean length (range)	–	859 (477–922)	811 (552–1050)	–
	Mean width (range)	–	33 (27–36)	29 (23–37)	–
More than 14	Mean length (range)	–	1022 (906–1130)	–	–
	Mean width (range)	–	29 (25–32)	–	–

*Abbreviations*: *na* not applicable (only single specimens available); -, no specimens available or no photographs taken during dissection process

## Discussion

More than 60 mosquito species are incriminated vectors of *Dirofilaria* spp., and several species have been examined under laboratory conditions for their potential vector competence by observing the development to the infective L3 stage. For *D. immitis* these include for example *Ae. aegypti* [40, 43–45], *Ae. albopictus* [46–48], *Ae. koreicus* [31], *Aedes vexans* (Meigen, 1830) [22] and *Aedes triseriatus* Say, 1823 [49], and for *D. repens* e.g. *Ae. aegypti* [45, 50–52], *Ae. albopictus* [46], *Ae. vexans*, *Aedes mariae* (Sergent & Sergent, 1903) [52], *Anopheles stephensi* Liston, 1901 [45], *Anopheles. maculipennis atroparvus* Wellcome, 1901 [45] and *Culex pipiens molestus* Forskål, 1775 [51].

To our knowledge, here we could demonstrate for the first time that field-collected *Ae. japonicus* are susceptible vectors for both *Dirofilaria* species. For example, the VEI for *D. immitis* was 66.7% which is distinctly higher than the corresponding values estimated for two other invasive *Aedes* species, *Ae. albopictus* and *Ae. koreicus* [31]. In addition, high abundances of *Ae. japonicus* have been reported from several introduction sites both in Europe and Northern America [32, 53–56]. Further, *Ae. japonicus* was shown to readily feed on mammals including humans and dogs [56]. Taken together, our findings suggest that there is an increased risk of *Dirofilaria* transmission in areas populated by this species. This is somewhat reminiscent to the situation in Italy where the establishment of *Ae. albopictus*, a suitable vector of *D. immitis*, changed the epidemiology of canine dirofilarioses (transmission in new areas, higher prevalences) [26, 27].

Interestingly, the laboratory colony of *Ae. japonicus* PEN was susceptible to *D. repens* but seemed refractory to *D. immitis*, i.e. no L3 larvae developed and reached the proboscis. Though we only had few blood-engorged females of this mosquito strain in this trial, this finding emphasizes again the need to carry out vector competence experiments with local and wild specimens to obtain relevant results. Unfortunately, the experiment cannot be repeated due to loss of the colony.

Additionally we could show that a laboratory colony of *Ae. geniculatus* derived from field-collected individuals [37] is an equally good vector for both *Dirofilaria* species. The univoltine *Ae. geniculatus* shares larval breeding sites such as tree holes with the invasive *Ae. japonicus* [37]. It generally occurs in low abundances but large numbers may be present in focal areas. Taking into account its aggressive mammophilic biting behaviour, *Ae. geniculatus* may contribute to local transmission cycles. Further, our established colony might be of value for further studies on host-pathogen interactions.

Constant temperatures of 27 °C are not realistic for central Europe, though the average temperature might reach this level during hot summer spells. Temperatures fluctuating over the day and between days are reality for the climate in central Europe, and further investigations will be done at more realistic fluctuating temperature regimes. Interestingly, daily temperature fluctuations accelerated pathogen development in the mosquito as compared to constant conditions with the same average temperature, as was shown with *Plasmodium* parasites, *D. immitis* and dengue viruses [57–59], and this was particularly observed under cool conditions which is of significance at the cooler margin of a suitable climate.

The developmental time from microfilariae to infectious L3 stage was in accordance with the predictions from the DDU formula. The experiments in this study were done at constant 27 °C; at this temperature, the development time is expected to be 10 days until the first observation of L3. However, even though first L3 larvae were observed within 10 days in *Ae. japonicus* and *Ae. geniculatus*, they tended to reach the proboscis only a few days later, and this has to be taken into account additionally when making a risk assessment.

In the first 2 dpi, *D. repens* L1 stages started to shorten in length, but remained at the same approximate width as microfilariae as compared to measurements described in literature [60]. After 3–6 days both *D. repens* and *D. immitis* had reached the typical sausage stage [39, 40] in *Ae. japonicus*, whereas they had only marginally shortened in the refractory *Ae. aegypti* IPNC, indicating that development did hardly take place in this mosquito species, and microfilariae most probably died in these first days post-inoculation. *Aedes aegypti* has been considered a rather unsuitable natural host for *D. immitis*, but a large variability of its susceptibility has been reported on many occasions [40, 43, 44, 61]. Both *D. immitis* and *D. repens* larvae started to elongate to L2 stage after 6 days at sizes comparable to what has been described earlier [40]. After 14 days and later, the infectious *D. immitis* L3 larvae reached their full length [40], with a lower size variation than described previously [22]. Interestingly, several larval stages of *D. repens* observed also in thorax and proboscis were shorter than previously described for L3 stages, and many of them were under 1000 μm. Previously, it was reported that the infectious stages reached lengths above 1000 μm [39].

Microscopy is a good and sensitive tool to observe the developmental stages of *Dirofilaria* spp. in mosquito samples. However, especially in the early stage of infection, additional positive specimens can be detected by PCR when low numbers of microfilariae originating from the infectious blood meals may be overlooked. In addition, PCRs remained positive until the end of the trials although development ceased at 5 dpi for *D. immitis* in *Ae. japonicus* PEN, and in *Ae. aegypti* IPNC for both filarial species. Thus, PCR positivity gives no clue whatsoever about infection rates and vector efficiency of any mosquito species, but can merely be of use in epidemiological studies to make a general assessment of occurrence of a filarial species in a certain area. This means that DNA reports from field-collected mosquitoes need to be very critically assessed.

We observed a large variation of microfilaria in the blood meals at 1 dpi (Tables 2, 3, 4, 5, 6, 7, 8 and 9). Furthermore, altogether only 75–94% of the engorged mosquitoes harboured microfilariae at all. This has to be taken into account additionally when making risk assessments. The calculations of VEI are based on the observed number of microfilariae at the beginning of the trial. Due to the low numbers of mosquitoes involved in some experiments, only between 1 and 3 mosquitoes could be used to determine the number of ingested microfilariae. Thus, the obtained VEI values have therefore to be treated with some caution. However, several mosquito species had L3 larvae in the proboscis and those can as such be seen as suitable vectors for the respective filarial species.

A crucial factor for vector competence and vector capacity is that mosquitoes need to survive the extrinsic incubation period in order to be able to transmit the pathogen to the next host. The results indicate that the parasite does not cause an overall higher mortality in the mosquitoes at the infection dose of 3000 mf/ml, which was chosen because it was shown to be suitable for such investigations in previous studies [31]. Higher mortalities with inocula containing higher microfilaraemiae were observed in various studies [31, 43, 48]. In endemic foci, very high microfilaraemiae can be observed (up to 70,000/ml) [31], and it was speculated that dogs with low microfilaraemiae might be the relevant reservoirs for *Dirofilaria* transmission [48].

## Conclusions

To our knowledge, field-collected *Ae. japonicus* were for the first time shown to be an efficient vector for both *D. immitis* and *D. repens*, indicating that this invasive and locally highly abundant species may contribute to a local transmission of filarial worms, as has been described for *Ae. albopictus* and *D. immitis* in Italy. Additionally, also the indigenous *Ae. geniculatus*, sharing the same larval breeding sites with *Ae. japonicus*, is a suitable vector for *Dirofilaria* spp. *Aedes japonicus* from a laboratory colony were refractory to *D. immits,* confirming the necessity to perform vector competence studies and risk assessments based on such studies with local mosquito populations. Our results further demonstrate that by DNA detection alone no reliable conclusions can be drawn with regard to the vector competence of a mosquito species.

## Abbreviations

DDU: *Dirofilaria* development unit
DIC: differential interference contrast
dpi: day post-infectionem
HDU: heartworm development unit
IR: infection rate
L1: first-stage larva
L2: second-stage larva
L3: third-stage larva
mf: microfilaria
PBS: phosphate buffered saline
VEI: vector efficiency index
